# Voluntary control of intracortical oscillations for reconfiguration of network activity

**DOI:** 10.1038/srep36255

**Published:** 2016-11-03

**Authors:** Juliana Corlier, Mario Valderrama, Miguel Navarrete, Katia Lehongre, Dominique Hasboun, Claude Adam, Hayat Belaid, Stéphane Clémenceau, Michel Baulac, Stéphane Charpier, Vincent Navarro, Michel Le Van Quyen

**Affiliations:** 1 Institut du Cerveau et de la Moelle Epinière, INSERM UMR S 1127, CNRS UMR 7225, Hôpital de la Pitié-Salpêtrière, Paris France; 2 Sorbonne University, UPMC-Paris 6, F-75005, Paris, France; 3 Department of Biomedical Engineering, Universidad de Los Andes, Bogotá D.C., Colombia; 4 Centre de NeuroImagerie de Recherche-CENIR, Institut du Cerveau et de la Moelle Epinière, UPMC-Paris 6, INSERM UMR S 1127 CNRS 7225, Hôpital Pitié-Salpêtrière, Paris, France; 5 AP–HP, GH Pitié-Salpêtrière, Epilepsy Unit, F-75013, Paris, France; 6 AP–HP, GH Pitié-Salpêtrière, Neurosurgery Department, F-75013, Paris, France

## Abstract

Voluntary control of oscillatory activity represents a key target in the self-regulation of brain function. Using a real-time closed-loop paradigm and simultaneous macro- and micro-electrode recordings, we studied the effects of self-induced intracortical oscillatory activity (4–8 Hz) in seven neurosurgical patients. Subjects learned to robustly and specifically induce oscillations in the target frequency, confirmed by increased oscillatory event density. We have found that the session-to-session variability in performance was explained by the functional long-range decoupling of the target area suggesting a training-induced network reorganization. Downstream effects on more local activities included progressive cross-frequency-coupling with gamma oscillations (30–120 Hz), and the dynamic modulation of neuronal firing rates and spike timing, indicating an improved temporal coordination of local circuits. These findings suggest that effects of voluntary control of intracortical oscillations can be exploited to specifically target plasticity processes to reconfigure network activity, with a particular relevance for memory function or skill acquisition.

An increasing number of studies provides astonishing evidence on the ability of humans and animals to control oscillatory rhythms, hemodynamic response, cellular activity or spike-related calcium signals upon presentation of sensory real-time feedback of the neural activity in question^1^,^2^,^3^,^4^,^5^,^6^,^7^. Cortical oscillations play a critical role in neural and cognitive function and represent thus an important target for voluntary control. It has been suggested that oscillations regulate network communication^8^, mediate long-range integration^9^, contribute to memory formation^10^ or to cognitive control^11^. Previous scalp electroencephalogram (EEG) neurofeedback studies have targeted theta, alpha, beta or sensorimotor rhythms, applying the training to boost attention^12^,^13^,^14^, memory^15^,^16^,^17^,^18^ or executive functions^19^,^20^,^21^. In a more engineering approach brain-computer-interfaces (BCI) based on EEG or electrocorticogram (ECoG) recordings are used to control a computer cursor, robotic limbs or restore motor function by modulating neural activity in motor-related areas^22^,^23^,^24^,^25^. The findings of these studies hold great promise for neural self-regulation that would permit restoration or enhancement of brain function in regular or clinical contexts. However, the fundamental physiological processes that mediate such voluntary control in the human brain are not often addressed and remain poorly understood, which would be necessary to capitalize on this technique^26^,^27^.

From the mechanistic point of view, neural oscillations represent large-scale cyclic modulations of extracellular potentials and local excitability of the network, which can attenuate or amplify faster, more local oscillations or neuronal discharges. By opposing temporal windows of integration, these self-organizing poles have the potential to coordinate local network activity and increase network communication and processing efficiency. Notably, it has been proposed that so called cross-frequency coupling may represent an electrophysiological signature of this improved coordination process during sensory and memory processes. Given that the communication between regions is influenced by the oscillatory coherence between the sender and the receiver^8^,^28^, changes in gamma oscillatory coherence, imposed by theta oscillatory phase, will selectively route information processing^29^,^30^,^31^,^32^,^33^.

Given this framework, it is likely that voluntarily induced slow oscillations can entrain higher levels of gamma coherence, that over time would give rise to more efficient communication between the local networks. Especially the theta rhythm, that is associated with high-level functions including cognitive control^34^,^35^,^36^ is an appropriate target for neural self-regulation that may facilitate downstream alterations even at a very local network and cellular level^27^. To study this question, we measured stereotactic electroencephalography (sEEG) and unit activity of seven neurosurgical patients while they learned to voluntarily induce theta oscillatory activity (4–8 Hz) at one target electrode and guided by the presentation of its visual real-time feedback. Although similar scalp-EEG studies have been conducted previously (e.g. ref. 21), intracortical recordings represent a particularly valuable data source to obtain mechanistic insight in neural function^37^, but also to better link human and animal findings on this subject. Combined depth- and micro-electrode recordings allowed us to examine activity from large-scale level until the very local neuronal activity of the human brain. We asked: i) Whether voluntary control of intracortical oscillations was possible ii) If so, what were the fundamental neural signatures of it and iii) whether we would detect downstream effects of induced oscillations on local circuit and cellular activity?

We have found, for the first time to our knowledge, that voluntary control of intracortical oscillations was possible in the human brain at various cortical locations. The leaning progress was expressed as an increased stability of the target signal and was associated with the functional decoupling of the target site within a larger network. This indicates that control of oscillations selectively potentiates and reorganizes existing circuits. Additionally, training induced a progressive increase in cross-frequency-coupling and dynamically modulated firing rates and spike timing, confirming that downstream effects of voluntary control of oscillations is mediated through changes in temporal network dynamics and can reach a very local level.

## Materials and Methods

### Participants and implantation procedure

Subject population consisted of 7 pharmacoresistant epilepsy patients (3 female, mean age 32.5, SD ± 12.7 years) undergoing pre-surgical evaluation at the Epilepsy unit at the Pitié-Salpêtrière Hospital. During presurgical evaluation, they were stereotactically implanted with depth electrodes (4–13 probes per patient) to localize the epileptogenic focus for possible resection. For each subject, 1–4 of the placed probes had additional 8 micro-wires emerging at the tip of the electrode into the grey matter. One subject had completed the training, but could not be studied due to an excess of interictal epileptic activity.

**Figure 1:**
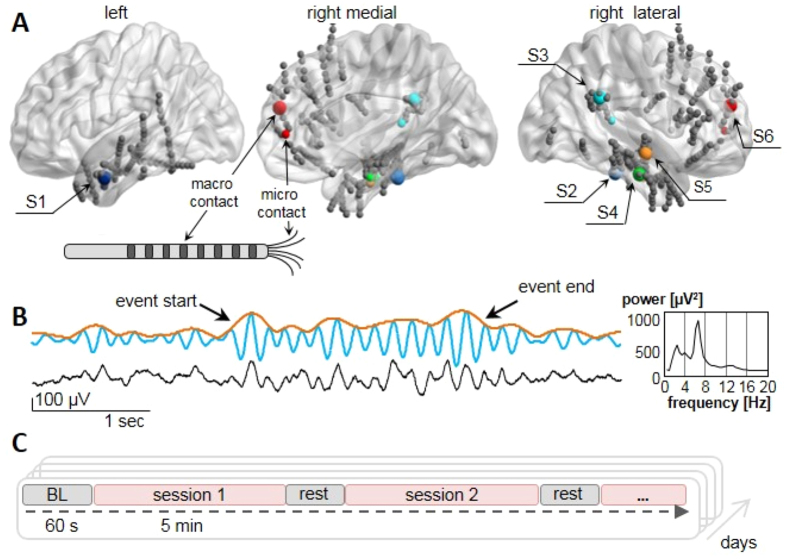
Training electrode positions, real-time signal processing and experimental paradigm. (**A**) Selected target macro-electrodes indicated by colored spheres and all other electrodes in grey. Smaller colored spheres show closest micro-contact to the target electrode. The color code is specific to different subjects and consistent across all figures. (**B**) Data treatment. Example of raw data trace showing a theta burst (black) with overlaid filtered signal (blue) and the corresponding envelope (orange). Arrows indicate the beginning and end of an automatically detected oscillatory event. Right panel shows a frequency spectrum of the data with a peak near 7 Hz. (**C**) Experimental procedure: a baseline period is followed by one or several 5-min sessions with intermittent pauses. Training is performed on 2–4 consecutive days.

The anatomical localization of the electrodes (Fig. 1A) was confirmed by the co-registration of the postoperative computed tomography scans with the preoperative 1, 5 Tesla MRI. The MNI coordinates of each contact were recovered automatically using the EpiLoc toolbox developed by the STIM (Stereotaxy: Techniques, Images, Models) (http://pf-stim.cricm.upmc.fr) facility at the Institut du Cerveau et de la Moelle Epinière and confirmed by visual inspection of postoperative MRI scans. Electrode positions were visualized with BrainNet Viewer (http://www.nitrc.org/projects/bnv/)^38^. All subjects had normal or corrected-to-normal vision and an IQ > 80 and gave their written, informed consent to participate in the study. The study was approved by the ethical committee of Pitié-Salpétrière Hospital (Comité Consultatif de Protection des Personnes participant à une Recherche Biomédicale, CPP). The experimental methods were carried out in accordance with the guidelines approved by the CPP.

### Electrophysiology

Stereotactic EEG (sEEG) was recorded with (depth) macro-electrodes (AdTech) of 4–12 platinum contacts 1 mm in diameter, with nickel-chromium wiring and polyurethane tubing. The macro-micro probes of Behnke-Fried type consisted of 8 platinum macro-contacts embedded on the surface of a polyurethane tube with a hollow lumen (diameter 1, 3 mm). Eight 40-μm platinum-iridium micro-wires were protruded 3–6 mm beyond the macro-electrode tip into the cerebral tissue (Fig. 1A, inset). During surgery, wires were trimmed to ensure they entered grey matter. Signals from macro- and micro-contacts were acquired simultaneously, at sampling rates of 4 kHz and 32 kHz respectively, with a 160-channel Atlas recording system (Neuralynx Inc., Tucson, AZ, Cheetah acquisition software). Bandpass filter settings for the macro- and micro-electrode recordings were 0.1–1000 Hz and 0.1–8000 Hz, respectively. Macro-contacts were referenced against the macro-channel providing the most flat and artifact-free sEEG signal. The reference for the micro-wires was chosen as a micro-electrode with no unit activity and a flat local-field potential (LFP). The distance between the conditioned target macro-electrode and the closest micro-electrode was for S1 to S6 in order (in mm): 35, 5, 33, 35, 37, 24.

### Closed-loop real-time processing

The real-time closed loop between the ongoing neural activity and screen display was realized using a TCP/IP connection between the EEG-recoding PC and a second laptop performing online analysis (Dell Precision M6700 Workstation). One target electrode was selected and followed throughout training. This electrode was chosen as a compromise between the maximal distance from sources of epileptic activity and proximity to micro-contacts. Target electrode positions for the 6 subjects were as follows (subject number:MNI coordinates/anatomical structure): S1: −57.7 −0.7 −28.1/left middle temporal gyrus (BA 21); S2: 40.2 −32.7 −25.1/parahippocampal gyrus (BA 36); S3: 66.3 −44.3 27.4/supramarginal gyrus (BA 40); S4: 55.5 −14.8 −26/inferior temporal gyrus (BA 20); S5: 57.6 −8.3 −4.6/middle temporal gyrus (BA 21); S6: 18.9 48.1 −0.2/anterior cingulate (BA 10). Distance between target electrode and the nearest micro-contract varied between 0.3–8 cm. To evaluate instantaneous theta magnitude, the envelope was measured as follows: 1) Received data were buffered in intervals of 400 ms, corresponding approximately to 3 oscillation cycles at 6 Hz for sufficient frequency resolution; 2) Digital bandpass filtering between 4–8 Hz was implemented through a zero-phase forward-backward digital infinite impulse response (IIR) type II Chebyshev filter^39^; 3) Hilbert transform was applied to this signal; 4) Absolute values corresponding to the amplitude were extracted for envelope estimation (Fig. 1B).

Visual feedback was presented on the laptop screen (graphics card NVIDIA Quadro K3000M, 17” screen, resolution 1920 × 1080 pixels) at a distance of 1 m from the subject. A spherical object was shown with vertical position on the screen varying as a function of the quantified mean theta envelope. 25 points were interpolated between sequential projections to reduce perceived jitter and provide a smooth movement. Continuous movement, which subjects said helped them interpret sphere movements, was further assured by averaging 4 bins (total 1.6 s). Online artifact control was implemented through a second threshold at >mean + 5 SD interrupting visual feedback until the values returned to a lower range. We verified that the visual display corresponded to changes in theta power by calculating the correlation between the movement of the spherical object and recorded theta activity within time windows of 10 seconds. Correlation values were not equal to 1 due to smoothing and online artifact control, but they were constant for all patients (average correlation values across sessions for S1 to S6 were: 0.39; 0.34; 0.51; 0.55; 0.44; 0.46).

### Training procedure

The training protocol included a short screening procedure and the actual training. During the screening the subjects explored different cognitive strategies to control the sphere movement. We suggested to try out several cognitive tasks, depending on the position of the target electrode and using a cognitive functional atlas database (www.linkrbrain.org. Tasks included visual, auditory, linguistic or spatial imagery, mathematical exercises, memory retrieval or executive functions. If a successful strategy could be identified, we asked the subjects to maintain it for the training condition.

Subsequently, three to six training sessions of 5 minutes each were conducted per day on 2–4 successive days, according to subjects’ willingness and capability to participate (session number: min = 7, max = 19, mean = 15.5, Fig. 1C). During the sessions subjects were asked to increase theta activity such that the sphere rose towards a target horizontal line on the screen. The thresholds determining this line were quantified as percentiles of a theta envelope distribution, measured over 60 s baseline before each session, with subjects in a relaxed but focused state^40^ and were adapted for each session to optimize training progress (used range: 70–95 percentiles). An additional feature of the feedback was the 30 s history in the form of a moving graph. We chose the 5-min long session duration instead of a few seconds-short trials after piloting for several reasons: 1) subjects reported a progressive build up in their control ability, such that frequent interruptions were disturbing this ‘tuning in’ process. 2) The history graph was a great help to subjects because it allowed to track fluctuations and better integrate different mental events. This graph was most informative over periods longer than a minute. 3) We aimed to create a training design that is as similar as possible to the normal process of any other skill acquisition, such that the learning would be more ecological and transferable to settings outside the laboratory. Reward points were accumulated across sessions for each threshold crossing to enhance motivation. An interview after each session was used to assess the utilized strategies, effort needed and subjective experience of voluntary control. Patients were asked to maintain their strategy after good performance or to change it if control was less efficient.

### Offline analysis

After training was completed, signals from all macro-contacts were re-referenced to a common average reference montage of at least 30 artifact-free channels for offline analysis. Possible muscle, movement or epileptic artifacts were screened visually across complete data sets. In total 403 macro-contacts were recorded, and signal from 326 artifact-free channels was used for further analysis (see Supplementary Figure S1 for individual electrode positions and Supplementary Table ST1 for anatomical regions). Electrical 50 Hz noise was suppressed by the same zero-phase forward-backward digital infinite impulse response (IIR) type II Chebyshev bandstop filter as used for real-time analysis. All analyses were implemented in MATLAB (The MathWorks).

### Evaluation of control performance

Throughout the article we will use the terms “control index” to refer to the degree of control in a specific session, and “learning index” to indicate progress in theta control of each subject across all sessions. The control index was defined the average theta envelop during sessions expressed as a percentage of the baseline mean value. Learning index was calculated as the slope of the linear, least-square fit of control indices for all sessions. Statistical significance of learning progress was tested by two measures: a) Pearson’s correlation coefficient between all control indices and temporal evolution and b) a paired-sample Student’s t-test comparing the control indices of the first and the last sessions for all subjects. The sample size for the t-test was n = 6, for the individual correlation analysis n = 19; 7; 15; 19; 15; 18 (number of sessions S1 to S6) and for group-wise correlation analysis n = 93 (sum of individual session numbers).

**Figure 2:**
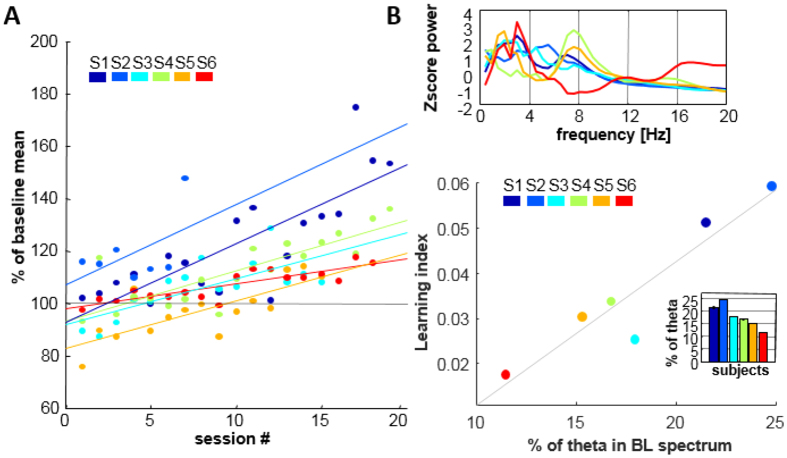
Training evolution, and baseline-performance relationship. (**A**) Evolution of control indices for all sessions and subjects. Each point represents the percentage of mean 4–8 Hz activity during that session relative to the baseline. The lines are least-square fits and the overall learning index is obtained from their slope. Subject color code as in Fig. 1A. (**B**) Upper panel: Proportion of 4–8 Hz activity in baseline spectrum before the first training session. Power units are transformed to z-score values to facilitate comparison across subjects. Lower panel: Proportion of 4–8 Hz activity within the 1–30 Hz spectrum of the initial baseline is strongly related to the overall learning index of every subject (r = 0.93**).

To calculate the individual power spectra, we first performed Gabor wavelet transform of the initial baseline condition and expressed the resulting matrix as z-scores (every value in the matrix was normalized by subtracting the frequency-wise average and dividing by the frequency-wise standard deviation). Next, spectra were obtained by averaging the normalized Gabor wavelet matrix along the temporal condition (for Fig. 2B).

### Oscillatory event detection

To analyze successful modulation periods in more detail, we extracted intervals of strong theta activity from all sessions. Automatic detection of these ‘oscillatory events’ was based on two criteria: amplitude threshold >mean + 1 SD of baseline (above the 68^th^ percentile of the baseline distribution), and a duration of at least 600 ms (about 4 theta cycles). Detection based on power or envelope yielded comparable results. Detected events were selected for further offline analysis from each subject, after visual inspection and removal of possible artifacts (number of selected events per subject, S1 to S6 in order: 886, 255, 384, 645, 255, 433, total 2858 events). The timing of identified events was consistent with threshold crossings detected online during experiments (data not shown).

### Spatial analysis of theta modulation

Spatial specificity of training was assessed by comparing control indices for trained vs. non-trained channels. Distances between the target and all other electrodes were first computed using Euclidian distance function, and then indices were calculated for nearest electrodes on the same probe.

Connectivity was analyzed using the approach of Adhikari and co-workers^41^. Pair-wise correlations of theta envelope time series during intervals of oscillatory events were calculated between the target and all other electrodes with Pearson’s correlation coefficient. Subsequently, binary connectivity matrices were generated by applying the threshold of r > 0.5.

### Regression analysis

Individual control indices for each session were regressed to a single linear predictor term, namely the number of connections per session. Models were generated with Matlab function ‘fitlm’ and resulting regression coefficients were used to fit the data.

### Theta-Gamma modulation index

Coupling of theta and gamma frequency was assessed in several stages. We first visually inspected time-frequency representations of all oscillatory events split into non-overlapping theta cycles aligned at the troughs. Time-frequency representations were calculated separately for each cycle and then averaged to preserve induced oscillatory phenomena. Gabor wavelet transform with a modulated Gaussian window was used to derive time-frequency decompositions^42^. Visual inspection suggested theta-gamma occurred at frequencies in the range 30–120 Hz. We then generated phase- and amplitude-time series for all sessions. Phase times series in the theta range were calculated with an IIR Chebyshev filter with a forward-backward filtering algorithm to avoid phase distortion^43^. Amplitude times series in the gamma range were extracted using Gabor wavelet transform. Theta-gamma modulation was then estimated using procedures as described^44^. To evaluate the coupling strength induced specifically through training, we calculated the MI as the difference between training and baseline modulation indices. Increase in coupling was assessed as correlation between time (individual session numbers) and MI.

### Firing rate and spike-field locking analysis

Spike sorting was performed offline with Wave_Clus software^45^ and verified with a second method Klustakwik^46^. We distinguished between multi-unit (MUA) and single-unit activity (SUA) using the criterion that less than 1% of spikes should show inter-spike-intervals (ISI) below 3 ms. We classed units recorded from the same channel but on different days as distinct cells. In the following we use the term neuron to refer to a putative multi- or single-unit. Binary data containing spike trains were converted to instantaneous firing rate using a Gaussian kernel convolution (kernel width 3 s). We classed neuronal activity during oscillatory events from all subjects (1641 events with spikes) according to increasing, decreasing or stable firing frequency and measure their distance to target electrode. A threshold of 40% was used to define changes in firing rate over 1000 ms before and after event onset (post-pre >40%: increase; post-pre <− 40%: decrease; −40% <post-pre <40%: stable). Spike-field locking was calculated from LFP phase-times series of the target electrode, extracted at the times of spiking, with distances between target zone and micro-electrodes ranging between 5 and 37 mm (see section Electrophysiology). Phase-locking was statistically evaluated with the Matlab toolbox for circular statistics^47^. We distinguished increasing, decreasing or sustained phase-locking, from Rayleigh Z-statistics for each session and their correlation with time (n = 9). Different patterns were assigned as for firing rate analysis (threshold at r = ± 0.4).

## Results

### Training performance is proportional to target rhythm strength before training

All subjects were able to reliably self-induce oscillatory activity in the target frequency and at the target cortical site. We quantified the degree of this control per session (control indices) as well as overall performance (learning indices) for each subject. All participants reached positive control indices which were strongly correlated with the temporal sequence of sessions (group level with r = 0.61, p < 0.001). While learning rapidity and degree of control differed, correlations of control indices with the temporal dimension were significant for 5 of 6 subjects. (S1, r = 0.8***; S2, r = 0.54^n.s^; S3, r = 0.73**; S4 = 0.85***; S5, r = 0.76***; S6, r = 0.85***, n = individual session number (see Material & Methods)). The utilized strategies were very individual and ranged from reciting the alphabet, thinking of a specific object, but also ‘breathing into the ball (visual feedback)’ or ‘talking to the ball’. Summary of all strategies is given in Supplementary Table 2. Most subjects reported an increasing effortlessness and a sense of control with training progress. For the entire subject group, mean theta activity during the final session was significantly higher than in the first (t-test: n = 6, p < 0.01). The increase in theta amplitude from baseline of the final session was on average 29% and maximally 53% (Fig. 2A). Although target sites were heterogenous among subjects, the broad spatial sampling shows the possibility of neural modulation through the cortex.

After showing that subjects were able to partially control theta oscillations, we asked whether training effects (learning indices) for this frequency were related to the baseline sEEG frequency spectrum prior to training start. The profile of the baseline frequency spectrum varied between patients, as expected from different target electrode locations (Fig. 2B, upper panel). We measured the prevalence of theta band oscillations [4–8 Hz] as the percentage of the spectral power integrated from 0–30 Hz in the baseline sEEG prior to the very first session (Fig. 2B, inset lower panel. We found the proportion of theta oscillations in the basal spectrum was strongly correlated with learning indices (r = 0.94, p < 0.01, Fig. 2B lower panel). Thus, the dominance of a rhythm in baseline conditions was indicative of the overall training performance.

### Voluntary control is manifested as increased oscillation event density

**Figure 3:**
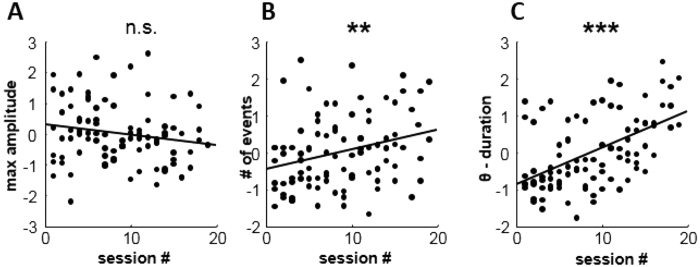
Theta control is manifested as increase in signal stability. (**A**) Maximal amplitude values of detected oscillatory did not change significantly during training. (**B**) Number of oscillatory events detected per session increased significantly with training progress. (**C**) The duration of oscillatory events increased significantly as proficiency improved. All values shown in A–C were z-score normalized to facilitate comparison across subjects.

We next attempted to identify how the increased mean power at theta frequencies was related to within- and across-session changes in oscillatory events. Three main features of detected theta band oscillatory events were measured: i) maximal amplitude, ii) occurrence frequency and iii) event duration. We found that maximal amplitude values were stable throughout the sessions (r = 0.18, n.s, Fig. 3A). However both the number and mean duration of theta events per session increased significantly with training (r = 0.29, p < 0.01; r = 0.52, p < 0.001, respectively; Fig. 3B,C). Thus, voluntary control of theta reflected increased signal stability with more frequent and longer theta oscillatory events (numbers of cycles per event) but with little change in amplitude of oscillations. This finding is in contrast to the possibility of amplitude increase as found for example for the alpha band^19^ or gamma band^1^.

### The predominant change occurs in the target frequency and cortical site

**Figure 4:**
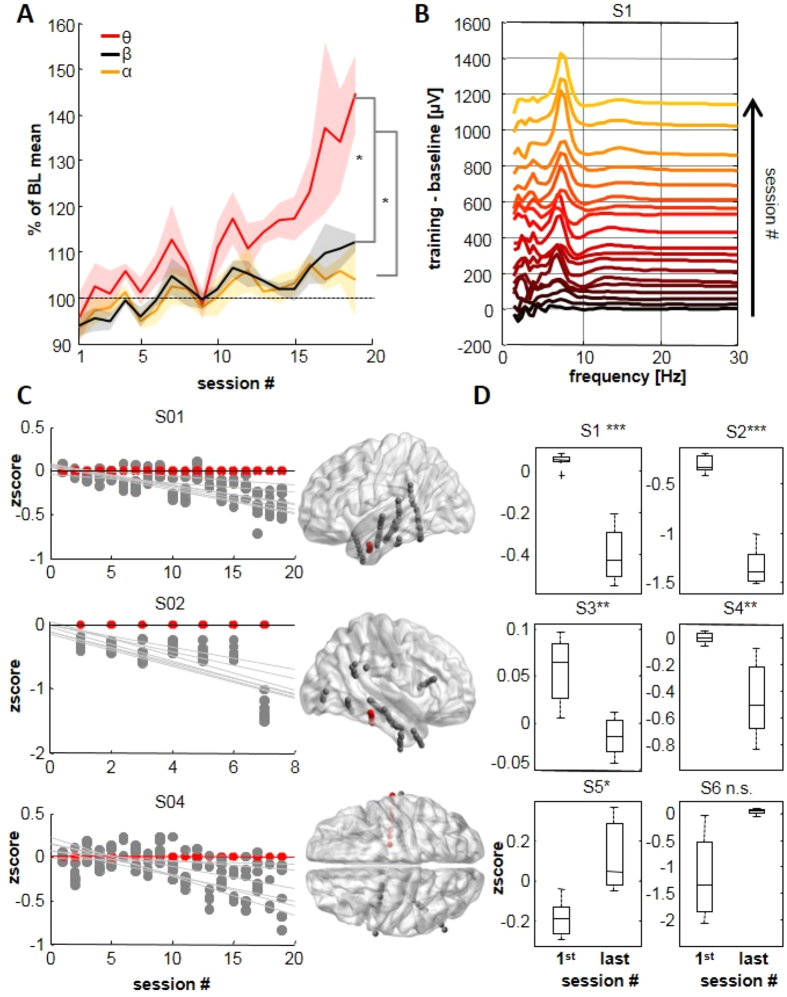
Frequency and spatial specificity of training. (**A**) Changes in theta, alpha and beta frequency bands for all subjects, all sessions. Solid lines are group means and shaded areas indicate standard errors. Changes in the theta band predominate. A significant difference between theta and beta/alpha emerged during the last session. (**B**) Frequency spectra from all sessions for S1. One line indicates the difference between one session and its baseline for the spectrum for 1–30 Hz. Traces are shifted vertically and color coded with initial spectra dark, advanced sessions in red and then yellow. The dominant spectral change occurs in the trained 4–8 Hz band. (**C**) For 3 subjects, comparison of 4–8 Hz activity between training electrode (red dots) and closest adjacent electrodes (grey dots). Values are represented as z-scores. Reduction in the scores from adjacent electrodes with training progress indicates that activity is diminished relative to training electrode. Right panels show the anatomical positions of compared electrodes (in red). (**D**) Evolution of the activity of closest adjacent electrodes between the first and the last sessions. Activity was reduced with respect to that of the target electrode for 4/6 subjects.

We compare concomitant changes in other frequency bands by computing control indices for delta [0.5–3 Hz], alpha [8–12 Hz], beta [12–25 Hz] and gamma [30–80 Hz] activities recorded at the target electrode for all patients. No power change across sessions was resolved for delta or gamma oscillations (n.s., Supplementary Figure 2). In contrast, alpha and beta band power increased significantly during repeated sessions (correlations between time and performance were r = 0.4 for alpha and r = 0.6 for beta, and t-test comparing first-last sessions (n = 6) was p = 0.035 and p = 0.027 for alpha and beta, respectively). Even so, the major change in the frequency spectrum occurred at theta frequencies, which was shown by comparing mean theta envelope values of the group from final sessions for theta compared to alpha and beta band power (Fig. 4A, p < 0.05, p-values were adjusted for multiple comparisons using the false discovery rate^48^. Figure 4B shows the dominance of the learning effect in the theta frequency band during sequential training sessions.

The spatial specificity of changes induced by training was examined by calculating control indices in the theta range for closest adjacent electrodes located on the same probe. As shown in Fig. 4C, activity on all electrodes reached initially similar levels. With training, a negative difference between the target and nearby electrodes emerged and increased progressively. T-tests of relative activity at nearby electrodes for first vs. the last session revealed a significant divergence from the target electrode in 4 of 6 subjects (Fig. 4D). Hence, the principal training-induced change was in the target frequency band and cortical site.

### Cortex-wide functional connectivity decreases with training and predicts learning progress

**Figure 5:**
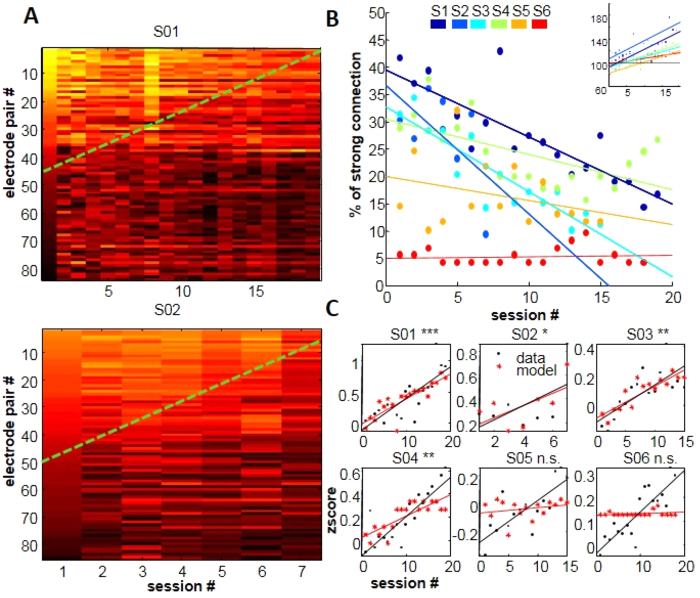
Reduction of theta functional connectivity with training progress. (**A**) Pair-wise correlations matrices between the training and all other electrodes from two subjects (upper and lower graphs). At training beginning strongly correlated electrode pairs are numerous (upper left corner) and decrease toward the end of training. Green dashed lines indicate a gradient of correlation decrease across sessions. (**B**) Summary of functional connectivity as the percentage of total electrode pair number, obtained from binary connectivity matrices with a threshold of r = 0.5. The degree of initial connectivity ranged between 5–45%. Connectivity was reduced by 0–20%. Please note the inversed subject ranking between network connectivity and training performance (small inset of repeated Fig. 2a in upper right corner). (**C**) Regression analysis and data modeling of individual control indices based on network connectivity from (**B**) Regression models were significant for 4/6 subjects, explaining between 30 and 67% of the overall performance variability. Models did not provide significant results for the two patients with lowest initial connectivity.

We next asked how training influenced large-scale spatial functional theta connectivity, by constructing a pair-wise measure of connectivity between the target and all other electrodes. Surprisingly, as shown in Fig. 5A, connectivity was decreasing as task performance improved during training. The mean number of connections fell from 25% to 13% across sessions (t-test first vs. last session p < 0.05, correlation between number of connections and time: r = −0.37***). Thus, theta oscillations at the target electrode become 29spatially autonomous (Fig. 5B). This decoupling was inversely related to the individual learning index as shown by the linear regression between control indices and the number of strong connections per session. For 4 of 6 subjects, linear regression models were significant and could explain between 31% and 63% of the individual variance of control indices (Fig. 5C). Importantly, both patients for which the prediction model did not turn out significant had also shown lowest initial connectivity and were the worst performing subject (S5 and S6). Thus, the reorganization of the large-scale network towards a functionally independent activity of the target area may play an important role during learning.

### Broadband gamma activities are increasingly modulated by oscillatory phase

**Figure 6:**
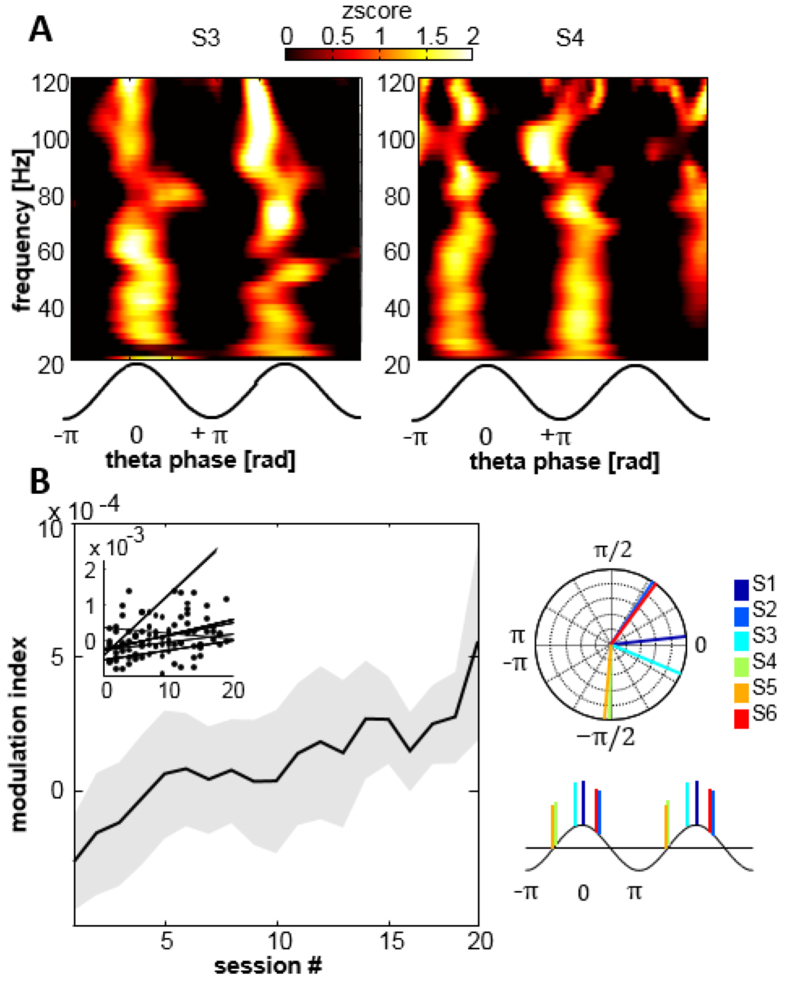
Theta-gamma coupling and multiscale interaction. (**A**) Average time-frequency representation of all theta cycles for 2 subjects. Theta-gamma modulation is present in the range of 30–120 Hz, occurring at different phases for different subjects. (**B**) Modulation index of theta-gamma phase-amplitude coupling is increasing significantly with training progress. Theta-events were concatenated within sessions to provide data length of at least 30 seconds.

Coupling between theta and gamma oscillations was suggested to increase in some learning paradigms^33^,^48^. We examined whether, during training, the phase of theta oscillations at the target electrode was linked to changes in power at higher frequencies. First, the time-frequency representation of aligned theta cycles was calculated to define the range of higher frequencies for further analysis (Fig. 6A for two subjects). Theta-gamma coupling was broadband from 30 to 160 Hz, with strongest modulation occurring in the range [30–120 Hz]. Theta phase associated with maximal gamma power varied between subjects, but was clustered in the depolarized (positive ascending/descending) part of the oscillatory cycle (phase in radians for S1 to S6 in order: 0.1; 0.96; −0.39; −1.58; −1.65; 0.91). We calculated the modulation index (MI) over the gamma range for all subjects and sessions relative to baseline (Fig. 6B). A significant positive correlation between MI and the training sessions (r = 0.3, p < 0.01) revealed a progressive increase in theta-gamma during this learning paradigm. To exclude the possibility of spurious higher detection of CFC through generally increased theta amplitudes, we calculated modulation indices in bins of 30 sec, showing that these binned MI-values do not correlate with theta amplitude (Supplementary Figure 3). Even so, it cannot be entirely excluded that the gradually increased number of theta bouts may lead to a higher theta-gamma coupling not visible in periods of 30 seconds.

### **Modulation of** cellular activity through theta oscillations

**Figure 7:**
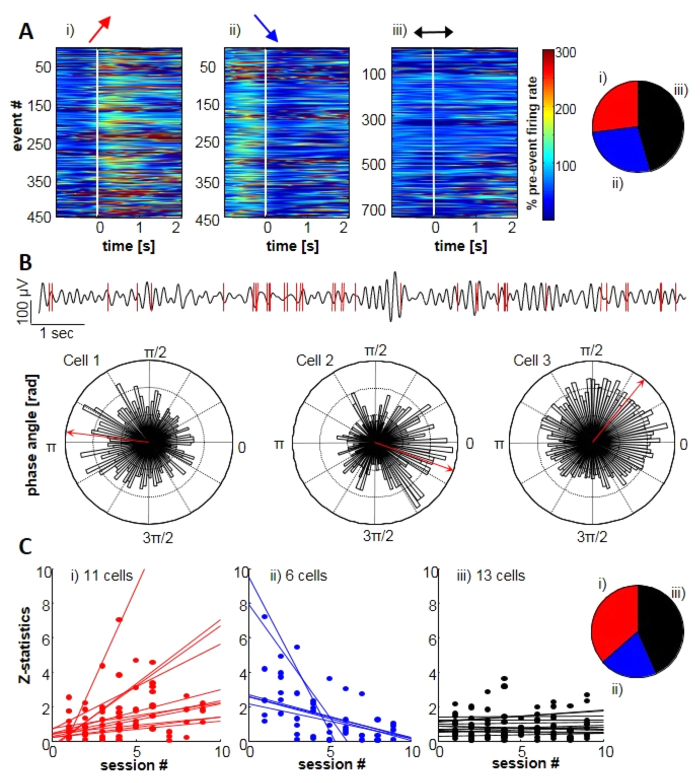
Firing rate changes and spike-field locking. (**A**) Classified detected events with increased (i, 27%), decreased (ii, 29%) or stable (iii, 46%) instantaneous firing rates at the moment when oscillatory event was detected (white vertical line). Right panel: Proportions of the three firing behaviors from 1641 events. (**B**) Upper panel: Comparison of filtered data (4–8 Hz, black) with spike detections of one cell (red) shows firing is grouped during descending phases an throughs of theta oscillation. Lower panel: example of spike locking to the LFP phase [4–8 Hz] for 3 cells. Red arrows represents the mean direction of the circular distribution. (**C**) Evolution of phase-locking during training. Different units showed trends of increase (i, 37%), decrease (ii, 20%) or no change (43%) of phase locking. Inset on the right summarizes the proportion of observed evolution patterns.

Finally, to explore possible training effects on unit activity recorded by micro-electrodes closest to the target electrode, we isolated 30 units (21 SUA, 9 MUA) from 5 subjects (See Supplementary Figure S4 for unit waveforms and firing rates). Analysis of global firing rates across sessions revealed no major changes relative to baseline (Supplementary Figure S5). However, the firing before and during oscillatory events was modulated differentially for different cells. Our analysis showed that firing rates increased for 27%, decreased for 29% or remained stable for (44%) of neurons during oscillatory events (Fig. 7A). We also examined the timing of spike firing with respect to the phase of ongoing theta oscillations recorded at the target electrode (Fig. 7B). Firing in 36% of cells was significantly locked to different phases of the local theta rhythm. Additionally, relations of unit firing to the phase of theta signal evolved across sessions: the level of unit-theta coupling was increased for 37% and decreased for 20% of units (Fig. 7C). In sum, this exploratory analysis showed that training of theta activities may have some influence on spiking activity. However, the effects are heterogenous and the small number of units makes a statistical analysis difficult, which makes future more rigorous testing necessary.

## Discussion

This is the first study, to our knowledge, to address voluntary control of theta oscillations by means of intracranial recordings in humans. These data show that human subjects can learn to endogenously induce intracortical oscillations at various cortical sites upon the presentation of visual real-time feedback, extending similar scalp-EEG findings^49^. Nevertheless, in contrast to conventional surface EEG, intracranial recordings have a high spatial resolution within a centimeter radius^37^, suggesting that voluntary up-regulation of theta activities is possible in quite restricted cortical domains (<1 cm). Additionally, while one previous scalp-EEG theta-training study showed a non-specific effect with co-increased beta and alpha rhythms^49^, successful intracranial modulation was specific in space and frequency and thus, training based on intracranial recordings appear to be of higher specificity.

Overall control ability was reflected in higher oscillatory event density across training sessions and proportional to the individual target frequency strength before training start. As control improved during learning, induced oscillatory activity at the target electrode became functionally decoupled from distant sites, which predicted the individual session-to-session performance variability. Locally, the training had a strong effect on broadband gamma oscillations that became increasingly phase-locked with theta activities in succeeding sessions and on neurons, that were modulated in firing rates and spike-field locking.

### Physiological signatures of voluntary control of oscillations

We found that individual training performance was proportional to the prevalence of the target rhythm before training. This supports previous proposals that acquisition of voluntary neural control is constrained by physiological neural properties^50^,^51^. It appears that if existing circuitry is pre-routed to generate a given rhythm, the voluntary control of this rhythm can be achieved more easily since it relies on existing inherent structures. This finding suggests that research and clinical training designs should ideally be customized according the individual spontaneous activity to achieve optimal training outcome and benefits. On the other hand, patients with small baseline values of the target rhythm have also shown an increase of oscillations to some extent. This provides some optimism that in the case of pathological absence of oscillations neurofeedback training may still produce moderate improvement.

Further, we found that successful modulation was manifested as a reinforcement of signal stability with (i.e. the increased density and duration of theta events) instead of supra-physiological boosts of oscillatory amplitude. Although other studies have found amplitude effects for other bands (e.g. refs 1 and 19), it appears not to be the case for the intracerebral theta rhythm. It follows that in general neural self-regulation can be based both on the increase of oscillatory magnitude (as hypothesized by ref. 52) and/or density. Such an enhanced signal stability, or signal to noise ratio, is reported during other related procedural processes such as attentional states^53^ or motor and abstract skill learning^54^,^55^,^56^. Indeed, previous work has shown that neurofeedback training can be used to increase cortical excitability and thus induce neuroplasticity in humans^57^ and reconfigure functional networks^58^. An alternative interpretation is that the physiological presence of theta oscillations is favorable for a general learning process as shown for the relationship between the presence of 2–8 Hz hippocampal rhythm and the conditioning rate^59^. However, this view would not explain effects detected outside the hippocampus. Since neuronal synchrony has been identified as a critical variable for the occurrence of Hebbian changes in synaptic efficacy^60^,^61^, repeatedly self-induced oscillatory activity in succeeding sessions should recruit neurons to population synchrony, so reinforcing the existing circuitry as they are constantly co-activated. In this context, long-term potentiation (LTP) was reported to be sensitive to the phase of the theta rhythm suggesting that this oscillation can act as a window permitting synaptic plasticity relevant for memory formation in humans and in animals^10^,^35^,^62^,^63^,^64^,^65^,^66^. Thus, it is likely that reinforced theta oscillations elicit a plastic process, which can stabilize the rhythm generating circuitry, further increasing theta elicitation probability in subsequent trials. Because there exist numerous studies testing for various behavioral benefits of neurofeedback techniques, and only few studies investigating their mechanisms, in the present investigation our guiding questions were of fundamental nature aiming to describe the physiological processes during neural self-regulation and not to examine behavioral effects. However, in the context of reported self-induced and systematic synchronization in the theta band and its potential consequence on plasticity, it would be compelling to include in future a cognitive paradigm to directly assess the consequence on memory function.

### Decreased long-range functional connectivity related to the learning progress

Our results show that functional connectivity between the target structure and surrounding frontal, temporal and parietal cortical sites is progressively reduced during learning. Additionally, this decoupling process explains individual variability in performance on a session-to-session basis, indicating its relevance in the learning process. Previous studies have suggested that disengagement of larger cortical networks may mark a transition from a deliberate towards a nearly automatized execution of a BCI task^67^. Indeed, the gradual reduction of connectivity was accompanied by subjects’ report of reduced effort and the increased sense of control suggesting that the initial recruitment of large cortical areas during top-down driven approach have transitioned into an adaptation process with more effortless execution. Importantly, two subjects with least initial network connectivity were among the poorer performer, which may be an indication that the initial cognitive effort reflected in the wide recruitment of networks is necessary to achieve good performance at later stages of the training. Evidence suggests that such training-related plasticity may be a shared feature with other forms of perceptual or motor procedural learning^26^,^67^,^68^,^69^. For instance, it has been shown that professional pianists show reduced recruitment of many extended areas like prefrontal or cingulate cortices in comparison to naive controls^70^. Thus, the functional decoupling of target area from a larger cortical networks may reflect a plastic process of network reorganization, which appears to be necessary during abstract skill acquisition^58^,^71^,^72^. However, this result stands in contrast to previously reported enhancement of connectivity within the salience network through neurofeedback training protocol of parietal alpha amplitude reduction^73^ and needs clarification in future investigation. Additionally, the possible effects of fatigue or routine should be controlled for, ideally using an active control group. A detailed examination of anatomical connectivity (for example measured with DTI) or a probabilistic functional tractography^74^ would be helpful to identify whether the changes in connectivity follow the functional networks.

From the theoretical perspective, during learning, changes at one site functionally segregate local circuits from activities of wider groups of neurons in terms of relative statistical independence from the whole system, which can lead to autonomous operation. The observed functional decoupling may thus reflect an increase in mathematical complexity of brain operations characterized by an integration/segregation balance in large-scale functional networks^75^, possibly indicating a larger capacity of information processing in the system. Although the exact role of these decreases in long-range functional coupling remains to be clarified, we propose that it could be a useful physiological marker of successful voluntary neural control or other ongoing learning processes.

### Downstream effects on local network dynamics

While previous studies have directly targeted the training of the gamma rhythm^1^,^24^,^76^ and multi-unit discharges^7^, here we show that higher oscillations can be also indirectly influenced through the training of slower rhythms and the accompanying increase in cross-frequency coupling (CFC). Task-dependent modulation of CFC in humans has been reported previously^33^, in particular associated with memory processes^77^,^78^. Here, we extend these observations by showing a progressive increase in theta-gamma coupling during training in voluntary control of theta oscillations with a preference for the depolarized phase of the oscillatory cycle. Even though a possible confound of CFC-increase due to more frequent theta occurrence cannot be entirely excluded, a gain in CFC would have functional implications in either case. If CFC occurs for longer times during training, it is likely to induce a clustering of activity at a particular phase and hence, an improved temporal coordination of neuronal population activity^79^. For instance, synchronized inputs of different presynaptic neurons will be more efficient in driving postsynaptic responses, while redundant signals between postsynaptic neurons will be decreased^53^. Importantly, through such selectivity of downstream targets, the signaling will be more efficient without increasing the size of recruited assemblies, which is consistent with our observation of increased cross-frequency coupling without increase in gamma amplitudes. At the same time, we detected no overall increase of firing rates, indicating that global contributions of neurons have not changed. Rather, neuronal discharges were boosted, dampened or sustained, probably depending on the contextual network activity^80^. The small number of units does not allow to draw any definite conclusions, but this pilot unit-analysis is encouraging for future testing of the idea that self-induced theta oscillations seem to have a temporally differential excitatory or inhibitory effects on single cells.

Additionally, previous work has shown that cross-frequency coupling imposes phase-locked spike generation at temporally precise time points in the theta-gamma cycles^81^, probably arising from the temporally modulated patterns of synaptic currents. This is in line with our observation of a significant phase preference of firing for a proportion of neurons, however we did not find a consistent increase of this coupling. The lack of a systematic changes both in firing rate changes or in spike-field locking could be due to the heterogenous implantation schemes and the varying distances between target and micro-electrodes, which should be examined more closely on a more uniform data set. Nevertheless, we believe that these observations provide a basis for more rigorous examination of potential downstream influences of theta oscillations on more local scales and can serve as a basis for future studies. If confirmed, such regulation of cellular activity can presumably facilitate spike-time-dependent plasticity mechanisms by altering synaptic weights and optimize pre- and postsynaptic communication and network coordination^82^,^83^. While one previous study has shown that neuronal discharge can be directly controlled through mental activity^7^, here we propose that neuronal self-regulation can occur also indirectly through the regulation of oscillatory rhythms, which may be easier to implement and exploit experimentally. In total, we suggest that in both ways, closed-loop training can be exploited as a means of self-induced plasticity regulation for specific network reconfiguration.

### Limitations of the study

There are several limitations of the present study. Most importantly, the anatomical heterogeneity and the small number of electrode locations (and thus training target) across subjects make it difficult to fully investigate the anatomical specificity of the response. A larger group of subject would allow studying voluntary control of oscillations across the whole cortex. A second concern is that our data were obtained from patients suffering from medically intractable epilepsy and some abnormal activities (such as interictal spikes or high-amplitude slow waves) constitute a source of signal artifacts. In our case, we minimized this contamination by selecting for recording sites that are virtually free from epileptiform activity. Still, there could be some significant alterations caused by the pathology or due to the medication. A third shortcoming arising from the scarcity of implanted subjects is the lack of a control group. Ideally a control group would perform training with a sham-feedback condition, which would allow to address questions of non-specific effects of repetition (e.g. fatigue or routine) and confirm whether the observed physiological changes of PAC and spike-field locking occur even in the absence of neurofeedback training. Lastly, a larger number of training sessions would be desirable to investigate the progression of learning curves over longer periods.

## Conclusions

Altogether, this work describes fundamental electrophysiological mechanisms of voluntary modulation of intracortical oscillations on multiple levels of brain activity. Our findings show that induced oscillations elicit changes in the large-scale network reorganization, and tune the temporal precision in local network dynamics, that both mark the successful learning process. We propose that targeting plasticity processes through neural self-regulation can allow to shape (ab-) normal brain activity with particular relevance for memory function and abstract skill acquisition.

## Additional Information

**How to cite this article**: Corlier, J. *et al.* Voluntary control of intracortical oscillations for reconfiguration of network activity. *Sci. Rep.* **6**, 36255; doi: 10.1038/srep36255 (2016).

**Publisher’s note:** Springer Nature remains neutral with regard to jurisdictional claims in published maps and institutional affiliations.

## Supplementary Material

Supplementary Information
