# Supplementary material for: Voluntary control of intracortical oscillations for reconfiguration of network activity
Source: Sci Rep. 2016 Nov 3;6:36255. doi: 10.1038/srep36255 (PMC5093688; doi:10.1038/srep36255)
Supplement: Supplementary Information [file srep36255-s1.pdf]

Supplementary Information

Voluntary control of intracortical oscillations for reconfiguration of network activity

Juliana Corlier-Bagdasaryan[1,2,CA], Mario Valderrama[3] , Miguel Navarrete[3], Katia Lehongre[4], Dominique Hasboun[1,2,5], Claude Adam[1,2,5], Hayat Belaid[1,2,6], Stéphane Clémenceau[1,2,6], Michel Baulac[1,2,5], Stéphane Charpier[1,2], Vincent Navarro[1,2,5], Michel Le Van Quyen[1,2,CA]

[1]Institut du Cerveau et de la Moelle Epinière, INSERM UMR S 1127, CNRS UMR 7225, Hôpital de la Pitié-Salpêtrière, Paris France

[2] Sorbonne University, UPMC - Paris 6, F-75005, Paris, France

[3] Department of Biomedical Engineering, Universidad de Los Andes, Bogotá D.C., Colombia

[4] Centre de NeuroImagerie de Recherche - CENIR, Institut du Cerveau et de la Moelle Epinière, UPMC-Paris 6, INSERM UMR S 1127 CNRS 7225, Hôpital Pitié-Salpêtrière, Paris, France

[5] AP–HP, GH Pitié-Salpêtrière, Epilepsy Unit, F-75013, Paris, France

[6] AP–HP, GH Pitié-Salpêtrière, Neurosurgery Department, F-75013, Paris, France

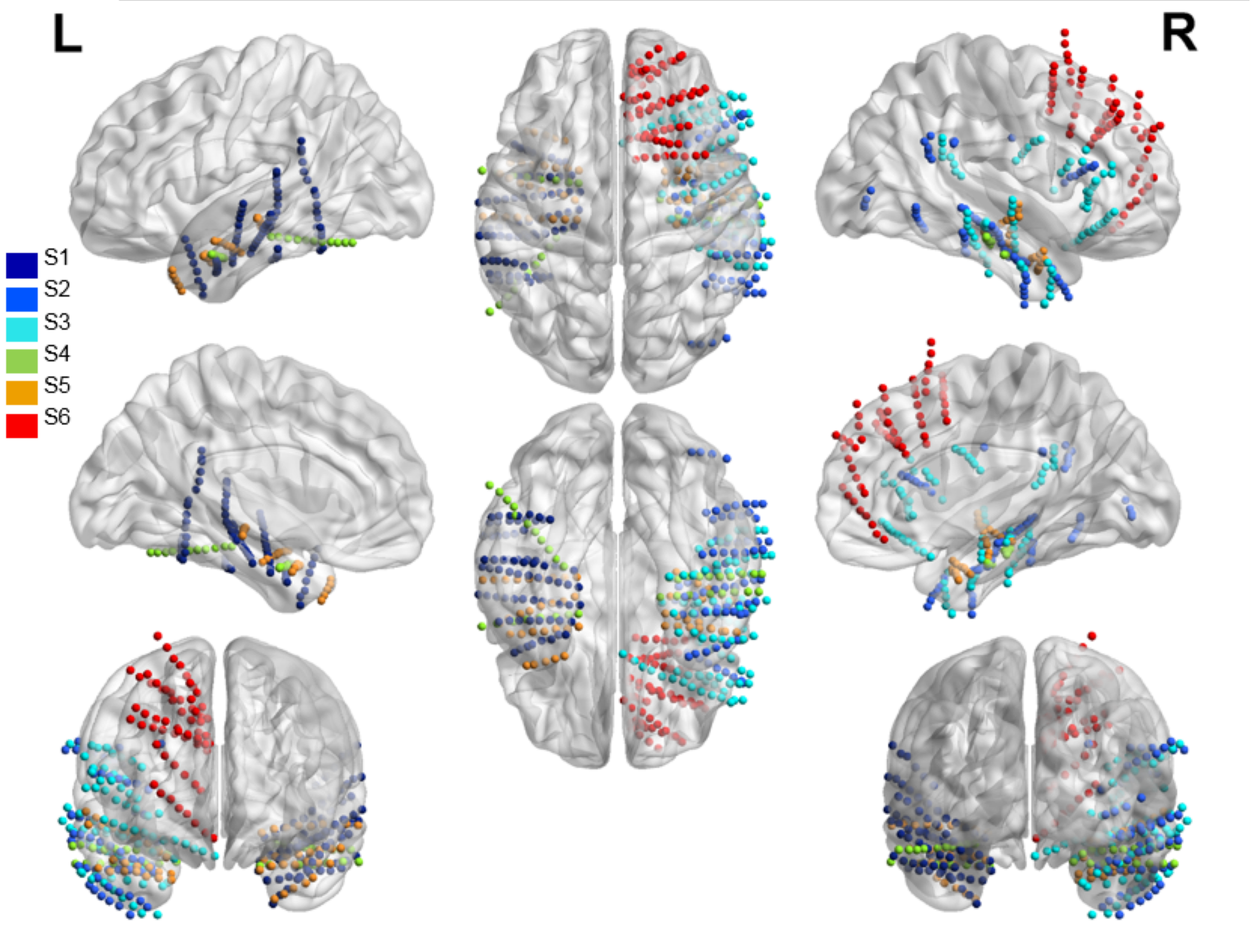

Supplementary Figure 1: Detailed individual implantation schemes

All electrode positions used for data analyses are represented for all subjects. Subject color code is consistent with other figures.

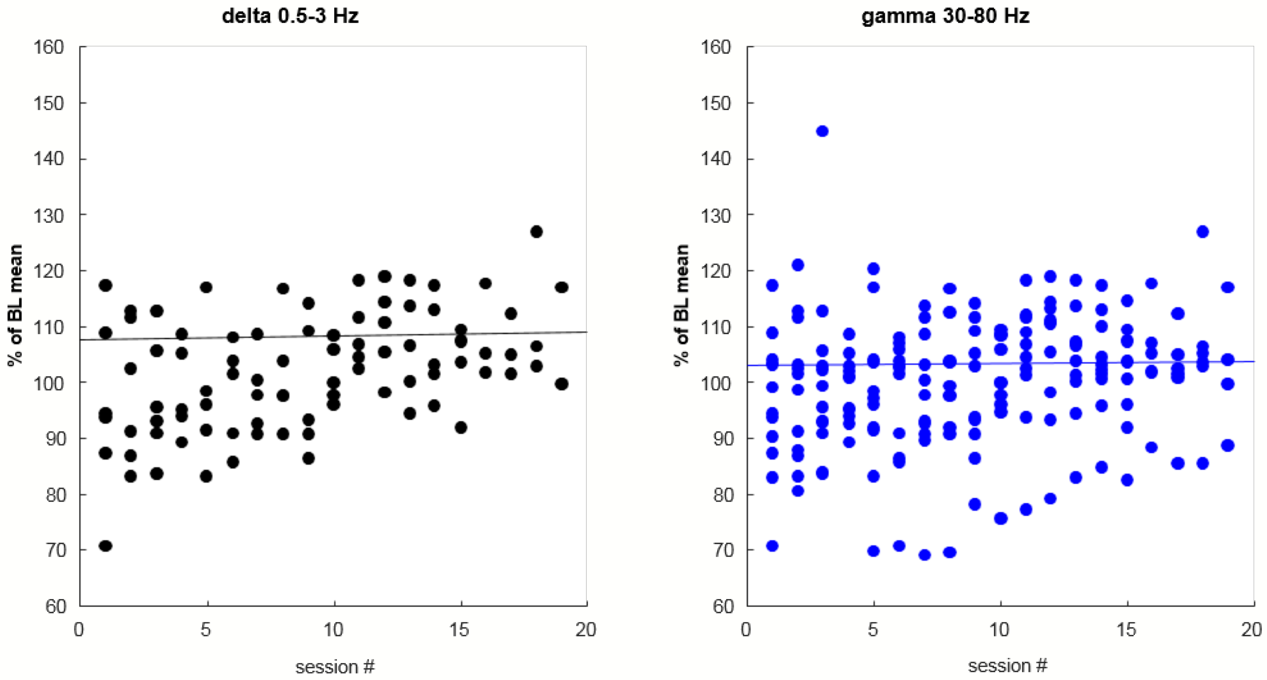

Supplementary Figure 2: Evolution of delta and gamma frequencies over the course of theta-training

No significant correlation is detected between envelop values of delta or gamma frequencies and time.

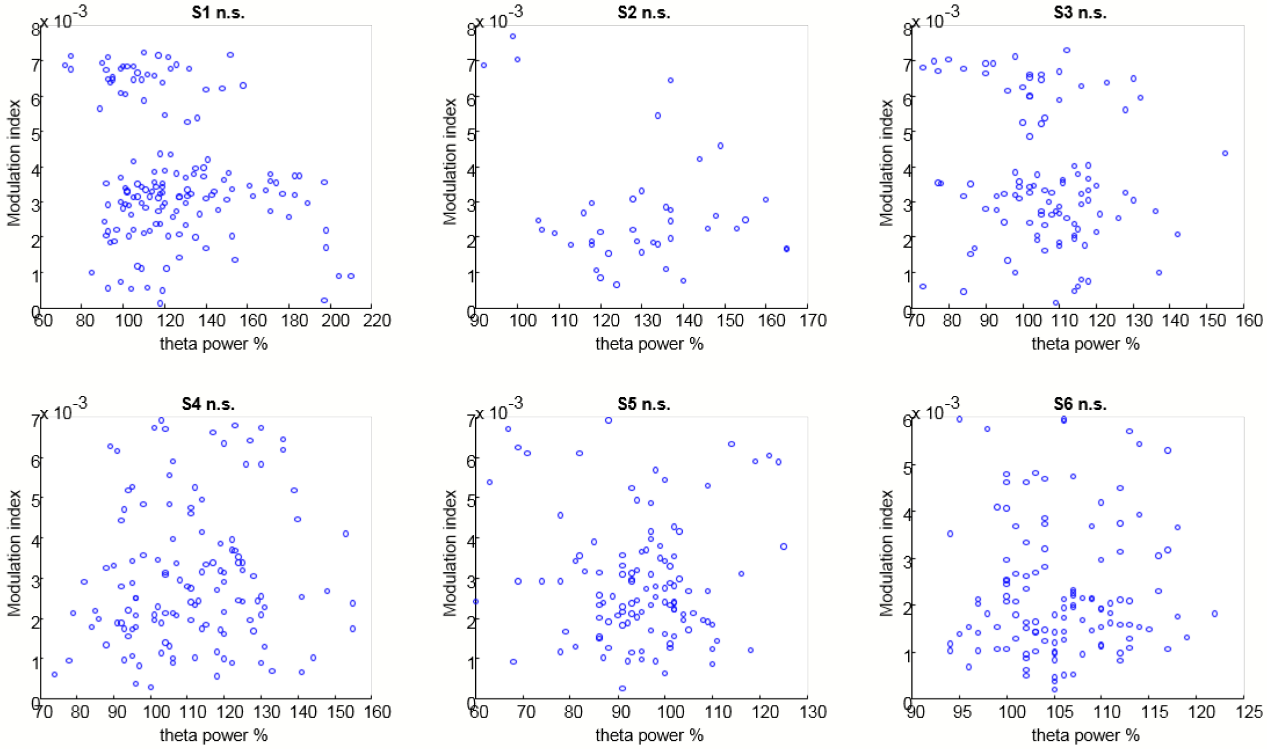

Supplementary Figure 3: Correlation between theta power and MI

Correlation between theta power (in % from baseline) and MI values, as calculated based on 30-seconds binned data segments, do not show any correlation for any of the subjects.

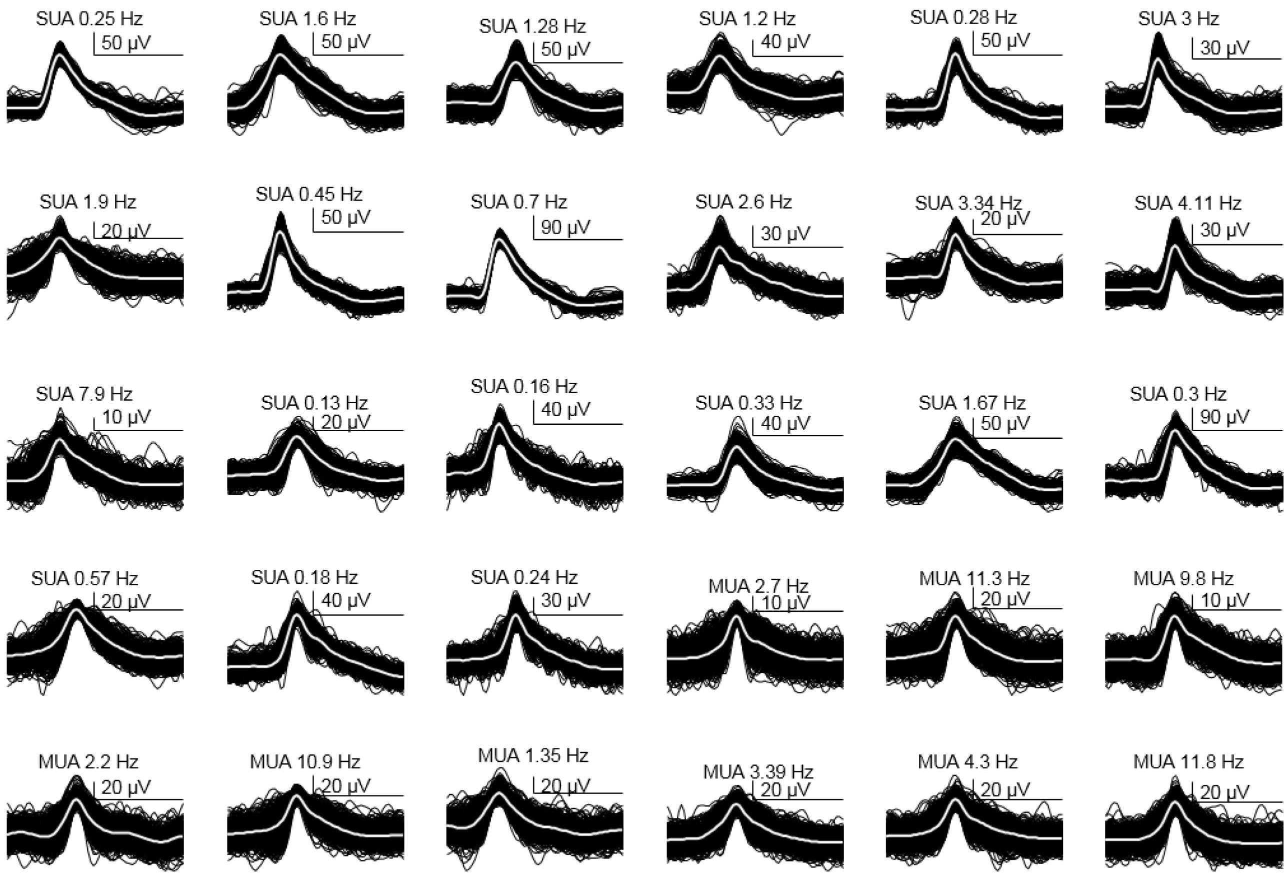

Supplementary Figure 4: SUA and MUA waveforms and mean firing rates

Representation of waveforms for all 21 SUA and 9 MUA. White trace shows average waveform. Horizontal scale bar represents 1 ms.

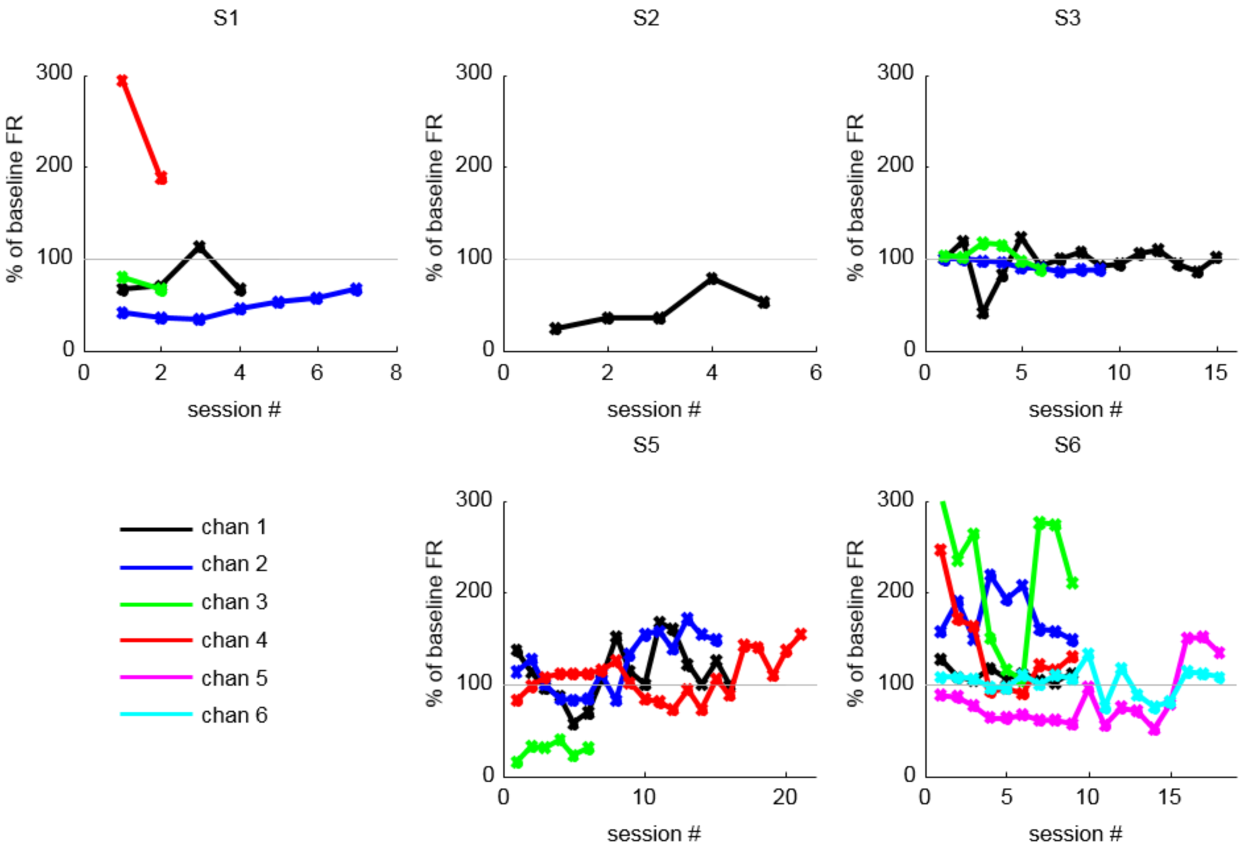

Supplementary Figure 5: Overall firing rates across sessions

Firing rates are represented in % from baseline activity. There here was no consistent change in overall firing rates across sessions. Different lines represent different channels. Note that units detected on the same channel, but on different days are considered as distinct units.

S1	S2	S3	S4	S5	S6
Left Fusiform Gyrus	Left Fusiform Gyrus	Left Fusiform Gyrus	Right Inferior Temporal Gyrus	Right Middle Temporal Gyrus	Right Anterior Cingulate
Left Inferior Parietal Lobule	Left Inferior Frontal Gyrus	Left Inferior Temporal Gyrus	Right Sub-Gyral	Right Superior Temporal Gyrus	Right Middle Frontal Gyrus
Left Inferior Temporal Gyrus	Left Inferior Parietal Lobule	Left Insula	Right Middle Temporal Gyrus	Right Uncus	Right Superior Frontal Gyrus
Left Middle Temporal Gyrus	Left Inferior Temporal Gyrus	Left Medial Frontal Gyrus	Right Parahippocampa Gyrus	Left Middle Temporal Gyrus	Right Superior Frontal Gyrus Post
Left Parahippocampa Gyrus	Left Insula	Left Middle Temporal Gyrus	Right Sub-Gyral	Left Parahippocampa Gyrus	
Left Temporal Sub-Gyral	Left Middle Occipital Gyrus	Left Parahippocampa Gyrus		Left Sub-Gyral	
Left Superior Temporal Gyrus	Left Middle Temporal Gyrus	Left Precentral Gyrus		Left Superior Temporal Gyrus	
	Left Parahippocampa Gyrus	Left Sub-Gyral			
	Left Sub-Gyral	Left Superior Temporal Gyrus			
	Left Supramarginal Gyrus	Left Limbic Lobe			

Supplementary Table 1: Anatomical labels of individual implanted regions

Anatomical regions of the entire electrode set. Number of electrodes recorded per region may vary between subjects.

subject #	sex	age	individual performance (linear slope of learning curve)	target site R = right L = left	strategy identified during screening	subsequent strategy I	subsequent strategy II	subsequent strategy III
S1	m	30	0.051	L middle temporal gyrus	–	» focus on relaxation, » imaging different body parts		
S2	m	26	0.059	R parahippo-campal gyrus	» mental rotation	» mental rotation » memories from childhood » semantic memory (animals)	» math exercises » perception of diff. body parts » focused & relaxed, not thinking about anything	» concentration, trying to reduce the space between ball and line, and feel it physically in the body
S3	f	55	0.025	L supramarginal gyrus	» birthday memory » reciting the alphabet backwards	» thinking of birthday » reciting the alphabet	» think of last vacations	» anticipation of going to vacation when the ball touches the line
S4	m	60	0.034	R inferior temporal gyrus	» thinking of an object (car)	» thinking of cars & family	» pushing the ball towards the line, » developing a sense of weight of ball in the body	
S5	f	61	0.030	R middle temporal gyrus	» attention of breath, to noises around	» concentration on the ball, » attention to environment » sense of pleasant feeling	» relaxing muscles, » breathing into the ball » imagining energy enters the ball pushing it	» imagining the ball becomes lighter » inducing a sense of wellbeing
S6	f	30	0.017	R anterior cigulate	» thinking of a close person	» imagining details of faces of close people and their emotion expression	» imagining oneself as a small girl » believing in own resources » thinking about the ball like of a friend	» emotion of goodwill to someone who is close (linked to the ball movement)

Supplementary Table 2: Cognitive strategies for voluntary control

The table presents individual cognitive strategies of all subjects as assessed after each session (strategies reported multiple times are presented only once). Gender, age, performance and implantation sites are also reported. Yellow marks strategies, which were derived from a personal interest of the subject (e.g. thinking of last vacations), and which was a particularly successful approach. In green, strategies are highlighted that were used by the subjects towards the second half of the training and represent a less well defined,'procedural' instead of clear-cut explicit tasks (e.g. 'breathing into the ball on the screen').
